# Effects of pre-analytical processes on blood samples used in metabolomics studies

**DOI:** 10.1007/s00216-015-8565-x

**Published:** 2015-03-04

**Authors:** Peiyuan Yin, Rainer Lehmann, Guowang Xu

**Affiliations:** 1 Key Laboratory of Separation Science for Analytical Chemistry, Dalian Institute of Chemical Physics, Chinese Academy of Sciences, Dalian, 116023 China; 2 Division of Clinical Chemistry and Pathobiochemistry (Central Laboratory), University Hospital Tübingen, 72076 Tübingen, Germany; 3 Inst. for Diabetes Research and Metabolic Diseases (IDM) of the Helmholtz Centre Munich at the University of Tübingen, 72076 Tübingen, Germany; 4 German Center for Diabetes Research (DZD), 72076 Tübingen, Germany

**Keywords:** Metabolomics, Preanalytical, Bias, Blood, Urine, Serum, Plasma, Liquid chromatography–mass spectrometry, Standard operation procedure, Biobank

## Abstract

Every day, analytical and bio-analytical chemists make sustained efforts to improve the sensitivity, specificity, robustness, and reproducibility of their methods. Especially in targeted and non-targeted profiling approaches, including metabolomics analysis, these objectives are not easy to achieve; however, robust and reproducible measurements and low coefficients of variation (CV) are crucial for successful metabolomics approaches. Nevertheless, all efforts from the analysts are in vain if the sample quality is poor, i.e. if preanalytical errors are made by the partner during sample collection. Preanalytical risks and errors are more common than expected, even when standard operating procedures (SOP) are used. This risk is particularly high in clinical studies, and poor sample quality may heavily bias the CV of the final analytical results, leading to disappointing outcomes of the study and consequently, although unjustified, to critical questions about the analytical performance of the approach from the partner who provided the samples. This review focuses on the preanalytical phase of liquid chromatography–mass spectrometry-driven metabolomics analysis of body fluids. Several important preanalytical factors that may seriously affect the profile of the investigated metabolome in body fluids, including factors before sample collection, blood drawing, subsequent handling of the whole blood (transportation), processing of plasma and serum, and inadequate conditions for sample storage, will be discussed. In addition, a detailed description of latent effects on the stability of the blood metabolome and a suggestion for a practical procedure to circumvent risks in the preanalytical phase will be given.

**Graphical Abstract:**
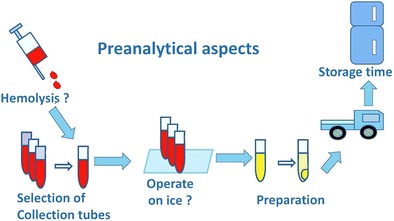
The procedures and potential problems in preanalytical aspects of metabolomics studies using blood samples. Bias in the preanalytical phase may lead to unwanted results in the subsequential studies

The procedures and potential problems in preanalytical aspects of metabolomics studies using blood samples. Bias in the preanalytical phase may lead to unwanted results in the subsequential studies

## Introduction

Metabolomics delineates biological phenotypes by profiling changes of endogenous metabolites [1]. In the past decade, metabolomics has been proved to be a valuable tool in translational medical research; it led to the detection of novel biomarkers for diagnosis, prognosis, and personalized medicine [2–5], and functional metabolomics studies elucidated novel pathomechanisms. Of note, although often described in a diagnostic context, functional biomarkers (lysophosphatidylcholines, amino acids, etc.) enable deeper insights into pathomechanisms but do not fulfill the criteria for diagnostic use, for example high diagnostic specificity. The most common biological specimens used in clinical metabolomic studies are body fluids and tissues [6]. Recent studies revealed the possibility of using tissue metabolomics as a reference for clinical resection, to distinguish malign from normal tissue [7]. For the discovery of novel diagnostic and/or functional biomarkers, noninvasive or less-invasive collected body fluids are usually used. As well as blood and urine, other body fluids including saliva [8–10], cerebrospinal fluid [11–13], feces [14–16], and follicular fluid [17, 18] are investigated in clinical metabolomics projects.

Both blood and urine are regarded as a “pool” of the metabolome. Urine contains many metabolic end products and metabolites that, e.g., originate from metabolized nutrients, drugs, and xenobiotics, etc. [19–22], whereas most metabolites in blood reflect the endogenous metabolites. Hence, the joint metabolomics analysis of blood and urine could provide complementary data reflecting the state of the whole system at a defined time point.

However, traditionally the collection and storage of blood samples in biobanks has been more common than collection of urine. One reason is that the 24 h collection of urine is cumbersome for the study subject and error-prone, and much more intrusive than the little effort necessary to draw blood. Furthermore, in spot urine the concentration of compounds is closely related to the individual intake of liquid, which makes adjustment, in particular of very diluted or highly concentrated urine, difficult. However, a limitation of blood samples is the risk of highly dynamic and pronounced changes of the metabolome in vitro, i.e. in the sample tube after blood drawing. Therefore, this pre-analytical phase needs to be tightly controlled and perfectly regulated during blood collection to avoid any negative effects on the metabolite pattern. This process is usually tightly regulated by a standard operation procedure (SOP) [23].

In complex clinical studies the blood collection is commonly described as the easy part. However, this easy part may greatly affect the sample quality and therefore the success of the study. Frequently, the actual quality of samples collected in a clinical study and used for metabolomics is an underestimated disadvantage. In particular, in multicenter studies it is still a major challenge to ensure that every hospital strictly follows the entire preanalytical procedure defined in an SOP. Care must also be taken that already existing SOPs are checked before the start of a metabolomics project for their general applicability, to ensure they are suitable for sample collection for omics approaches. For example, with the objective of achieving consistent conditions between different hospitals, a guideline for the collection of blood samples for a breast-cancer clinical trial recommended completing the process, from phlebotomy to storage of the samples in a freezer, within 8 h [24]. However, after vein puncture the blood cells should be removed and the plasma or serum preserved in a refrigerator as soon as possible (for details see section “The handling of whole blood after drawing”). The ultimate objective for blood collection in the context of omics studies (and many others) is to define the safety margin for sample handling after blood drawing, including establishing a procedure to ensure sample quality [25, 26]. However, frequently the daily clinical routine or individual local limitations, for example no direct access to a centrifuge at the place where the blood is drawn, impede the fulfillment of perfect pre-analytical procedures. Hence compromises need to be made between perfect preanalytical processing, the feasibility in the clinical study, and possible preanalytical effects on analytes. Accordingly, the generation of a feasible SOP is a challenging task which can only be realized through close collaboration of the clinical scientists and pre-analytical experts.

This review will report critical aspects of the preanalytical phase for clinical metabolomics studies, with a special focus on the sampling of blood. Generalized preanalytical aspects including relevant factors before sample collection, material preparation (tubes etc.), and collection, transportation, and storage of blood specimens are discussed. Practical suggestions and recommendations to circumvent and avoid preanalytical problems will be presented.

## Metabolomics and clinical applications

The measurement of blood metabolites in clinical settings has a long history. For decades many different metabolites, including glucose, creatinine, acylcarnitines, urea, uric acid, ammonia, bilirubin, bile acids, cholesterol, amino acids, fatty acids, and many others, have been used in clinical chemical laboratories to investigate the state of health [27, 28]. Now, by use of metabolomics, thousands of endogenous and exogenous compounds in blood with different chemical and physical properties and biological stabilities can be profiled at the same time [29]. Complementary targeted and non-targeted-analysis metabolomics is an important technique for systems biology and translational medicine, in particular in combination with transcriptomics and/or proteomics investigations [30, 31].

An important aspect of clinical metabolomics is the elucidation of biomarkers for the diagnosis and prognosis of disease, and the subsequent investigation of their function in projects of translational medicine. Early detection of disease is of great importance for the prognosis of the patient, especially for chronic and serious diseases including cancer, diabetes, and cardiovascular disease [32–35]. A common strategy for the identification of novel biomarkers includes two steps: discovery and validation. In the discovery step, profiling of a broad range of metabolites in a small set of samples is performed by a non-targeted metabolomics approach with the objective of detecting potential biomarkers [36]. In the subsequent validation step, targeted measurement of the elucidated potential biomarkers is applied to a large sample set to study the diagnostic power (sensitivity, specificity, robustness, etc.). This strategy is also suitable for the investigation of prognostic and other types of biomarker [36, 37].

**Fig. 1:**
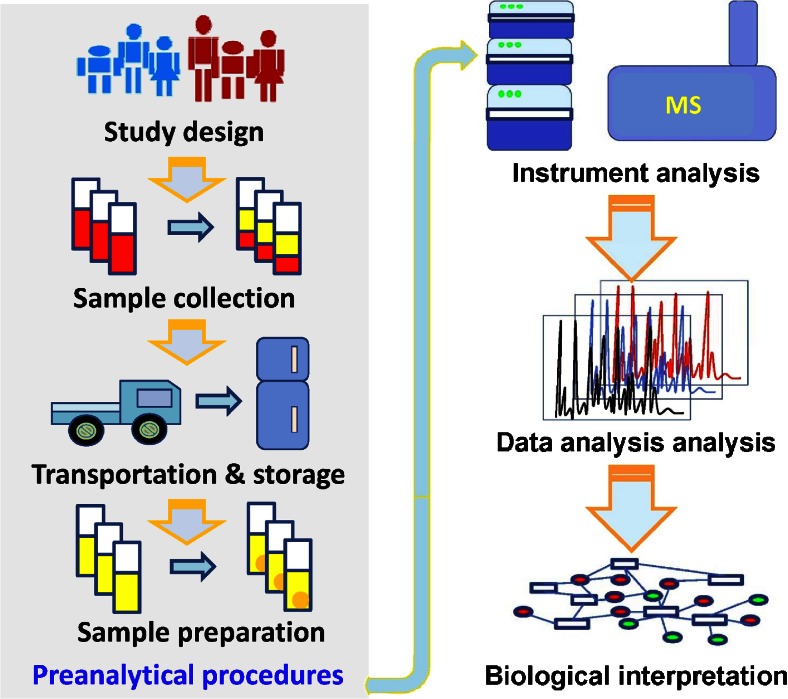
Scheme of the main steps in a clinical metabolomics study. The pre-analytical procedures are given on the *left-hand* side

Figure 1 illustrates the steps of a non-targeted clinical metabolomics study from the preanalytical phase to the final interpretation of the data:

1. Design the experiment (see section “Preparation before blood collection”). This step should include joint discussion by medical researchers, preanalytical experts, and analytical (bio)chemists to draft and define the SOPs. Because samples are usually collected and temporarily stored by nurses, medical students, or doctors, this point requires intensive discussion to adapt and optimize the SOP, focused on the local practicability in accordance with the preanalytical quality management to ensure sample quality. Further steps at this stage are designing the study, performing biostatistics defining the number of samples needed, drafting a questionnaire for the study subjects (if necessary), and finally applying for ethical clearance.
2. Collection of biological samples according to the SOP (please find a recommendation in the section “Preanalytical SOP for blood in metabolomics”).
3. Preparation of samples. Sample preparation includes protein removal and metabolite extraction (see section “Sample pretreatment for mass-spectrometry analysis”). This is a crucial step in the analytical process, because the categories of metabolite to be measured are specified by this pretreatment step [38]. Steps 1–3 are so-called preanalytical steps.
4. Instrumental analysis. Mass spectrometry (MS) and nuclear magnetic resonance (NMR) are the commonly used methods.
5. Data processing. The data collected from the instrument are usually multidimensional and include interference from chemical noise. The data-processing procedure commonly includes data reduction, denoising, metabolite extraction, and alignment [39].
6. Interpretation of the results. In this step, the chemical structures of the potential biomarkers should be identified, especially for the MS-based analytical techniques. If systems-biology approaches were used and multi-omics data need to be combined and evaluated for pathway visualization and enrichment, sophisticated software tools, for example InCroMAP, are needed [40].

It is important to note that an error-prone preanalytical phase unavoidably leads to poor, possibly misleading results; in such cases all efforts by the analytical (bio)chemist to enhance accuracy, sensitivity, and specificity in the analytical phase cannot compensate for the preanalytical errors and are consequently in vain and a waste of time.

## Preparation before blood collection

The metabolite pattern in blood is a tightly controlled homeostatic system, but a variety of physiological conditions and exogenous factors may lead to dynamic changes. As well as the possible substantial effects of the preanalytical phase [25], the composition of the blood metabolome is also affected by multiple intrinsic and extrinsic factors, including circadian and physiological rhythm [41], diet [21], exercise [42], drugs [43], and others [44] (Table 1). For this reason, well-considered preparation of the study subjects is needed before sample collection for metabolomics studies.

**Table 1:** Factors affecting the outcome of metabolomics studies that should be considered before sample collection, i.e. in the study design and sample-collection procedure

Factor	Recommendations	Main metabolites and references (selected examples)
Sex^a^	Match the distribution of sex	Lipids [45, 46], orthophosphate, α-tocopherol, creatinine, DHEA-S [47], cholesterol [44]
Age	Match the distribution of age	Amino acids [48], isocitrate, succinate, malate, lactate, etc. [47]
Body mass index (BMI)	Refer to BMI before the enrollment of participants, and match for BMI	BCAA [49–51], lipids, steroids [52]
Fasting and feeding	12 h fasting (usually overnight)	Essential amino acids and acylcarnitines [53], triglycerides and homocysteine [54]
Circadian rhythm	Keep normal biorhythm; collect samples at the same time point (usually in the morning); take circadian rhythm into account if metabolite concentrations follow a rhythm	Lipids [55], BCAA, lactate [56], bilirubin, cortisol [56, 57]
Exercise; stress	Avoid unaccustomed physical activity; avoid stress before drawing blood	Lactate, free fatty acids, glucose, amino acids and acylcarnitines [53, 58], uric acid [44], creatinine and many others
Drugs and/or nutritional supplements (e.g. vitamins, amino acids)	At least 12 h abstention, preferably 24 h or longer	–

^a^Sex-specific differences in metabolite concentrations should always be assumed until proved otherwise

Sex difference is a relevant and important factor in metabolomics studies [45, 59] (Table 1). Ishikawa et al. studied the plasma-lipid profiles of men and women of different ages, and reported a bigger difference between older males and females [45, 46]. Lawton et al. measured 300 compounds in 269 individuals and found that the concentrations of more than 100 metabolites were related to age [47]. Hence, to avoid age-related bias in metabolomics, results-matching for age is recommended. BMI is also an important factor in metabolomics studies. Morris et al. summarized the association between BMI and metabolomics profiles [49]. With the exception of lipids, branch-chain amino acids (BCAA) were reported to be the metabolites most closely related to BMI [49]. Consequently, it is of great importance for the study design to take into account age, sex, and BMI, and kidney and liver function etc., and to match the subjects regarding such factors (Table 1).

Fasting, according to clinical regulations, is recommended before sample collection, because the metabolite profile in blood undergoes dynamic changes during a period of several hours after meals [53, 60]. For example, 3 and 5 h postprandial the levels of essential amino acids and acylcarnitines change significantly [53]. It is therefore necessary to establish which interval without food intake is suitable. For the oral-glucose tolerance test at least 8 h and for the measurement of triglycerides and homocysteine 9–12 h fasting is recommended [54]. In Table 1 we recommend 12 h fasting, on the basis of long-established recommendations for medical examination of metabolic functions which have been revealed to be also suitable for metabolomics studies [53, 54]. Furthermore, a study by Winnike et al. revealed that one-day dietary standardization before sample collection can normalize the effect of food intake [61].

Physical exercise, stress, and several lifestyle aspects are also important factors affecting the blood metabolome and should be avoided before blood collection. Exercise may lead to increases in levels of lactate, some amino acids, and acylcarnitines, and to decrease of fatty acids etc. [53, 58, 62]. Furthermore, lifestyle factors including smoking also led to a clear separation of the metabolic profiles in blood in a comparison of cigarette smokers and nonsmokers [63]. Thus, matching lifestyles of the participants of a study on the basis of the information in a questionnaire could be an effective strategy to minimize avoidable bias of the results.

Blood is not only collected in the morning, but sometimes also at other times of day, e.g. in huge epidemiological projects, for example national cohort studies. Therefore the question arises as to whether the circadian rhythm affects the blood. In the results of Ang et al., 19 % (203/1069) of metabolite changes had significant time-of-day differences [56]; 34 affected metabolites were identified, including carnitines, LPCs, LPEs, bilirubin, cortisol, and amino acids [56]. Dallmann et al. reported that lipids are closely associated with the biorhythm, which may be, at least to some extent, related to food intake [64]. Branched amino acids and lactate were also found to be affected by the biorhythm [64]. Hence, the light–dark cycle, sleep–awake rhythm, time point, composition of last food intake, etc. should also be taken in account, and studies should avoid mixing samples collected at different times of day, at least for metabolomics studies.

Other very common, important factors affecting the metabolism, and the outcome of metabolomics studies, are drugs. The intake of drugs should be established in the questionnaire, because otherwise metabolic effects of drugs may be misinterpreted as findings relevant to the experiment. In particular, in studies investigating individuals aged above 45 years the daily use of drugs is commonly a factor. For example, new and unexpected findings in a lipidomics study may also be caused by a mismatch in the study population of subjects taking statins. Other, often less-considered factors affecting the metabolome are dietary supplements, including fish-oil capsules, multivitamin preparations, amino-acid and protein shakes, etc. Supplements are often less reported in questionnaires by the study subjects because these compounds are not drugs and are not regarded as substances affecting the results of the study. However, the effects of these supplements on metabolomics results may always be present in human studies, and the possible misinterpretation of the acquired data is not to be underestimated. Therefore, in the study questionnaire dietary supplements and drugs must be included and this information should be also reported to the analytical chemists.

Table 1 summarizes factors affecting the outcome of metabolomics studies that should be considered before sample collection, i.e. in the study design and sample-collection procedure, to avoid misleading results and the reporting of useless biomarkers.

## The selection of blood-collection tubes

The development of modern analytical MS instruments has enabled highly sensitive analysis of metabolites, but this is associated with a higher sensitivity to chemical noise signals. In comparison with other interfaces, the commonly used electrospray ion source is more sensitive to matrix effects [65].

**Fig. 2:**
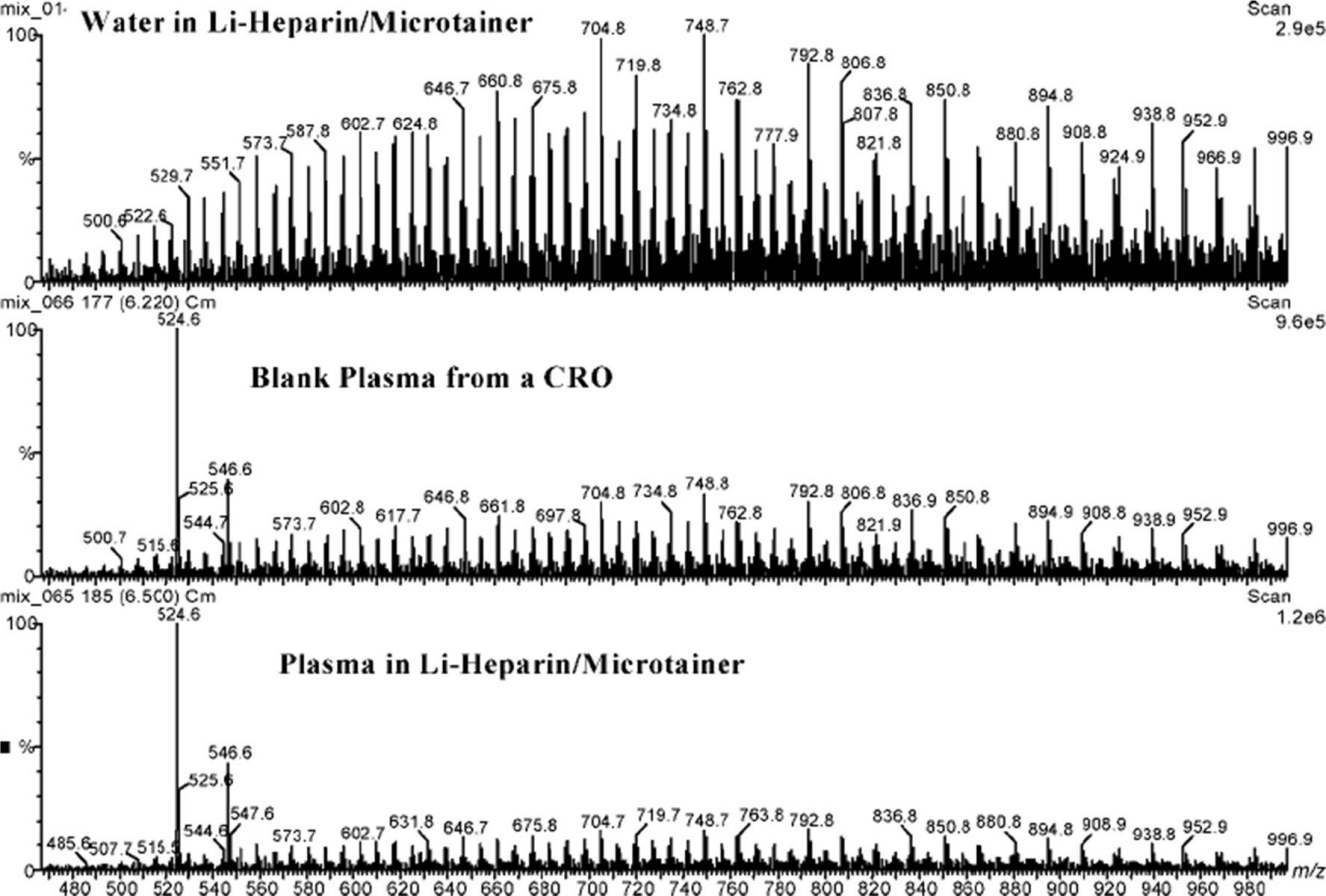
The mass spectrum of polymers shed from Li-heparin Microtainer plastic tubes (Reprint from RCM (2003)17 (1):97–103, license number: 3485190734889). When pure water, blank plasma, and plasma were added to the tubes, typical mass-spectrum patterns could be observed which may cause significant matrix effects on MS

The blood-collection tube could be a major source of chemical noise by introducing exogenous interferences into blood samples [25]. Characteristic patterns of tube-dependent chemical noise originating from plastic polymers in lithium-heparin sample-collection tubes are shown in Fig. 2. These interferences lead to substantial signal suppression of metabolite ion masses. On the other hand, Korfmacher et al. reported that Li^+^ increases the ionization efficiency of many metabolites [65]; however, the signals of polymers may also be increased by the use of Li-heparin Microtainer tubes (Fig. 2). In our recent study we used plastic sample-collection tubes with different anticoagulants, and also detected a strong chemical noise in the Li-heparin tubes [25]. Glass collection tubes may eliminate this problem but, justifiably, they are not commonly used in the clinic. In conclusion, great caution in the planning phase of a clinical metabolomics study is advisible when selecting the sample tubes. A pretest is absolutely mandatory, and the tubes of choice must be mandated in the SOP.

As well as the collection of whole-blood samples in tubes, the collection of a dried blood spot (DBS) on a filter paper is a convenient method with a long history in the clinic. DBS is a standard sample matrix in newborn screening to profile for inherited diseases, because only a small volume of blood per spot from heel prick is needed (<50 μL) [66]. Targeted metabolomics is the method most often used to analyze DBS [67, 68]. DBS and the following solvent extraction are regarded as one of the least invasive and most efficient strategies for whole-blood-sample collection and preparation in targeted metabolomics [67]. DBS has also been used in some nontargeted metabolomics studies [69, 70] and has been considered as an alternative specimen. Another crucial point is the decision to use either plasma or serum, which entail the use of different blood-collection tubes. Both serum and plasma are widely used in metabolomics studies, but there is an ongoing debate regarding which sample material is better suited for metabolomics. In this context scientists should be aware that metabolic profiles of plasma and serum are different per se, at least to some extent (Table 2). Plasma-sample collectors have the great advantage that the samples can be put at once into ice water. This avoids adverse effects of exposure of the samples to room temperature. In contrast, blood intended for the generation of serum needs to clot at room temperature for a defined time, usually >30 min (for details see section “The handling of whole blood after drawing”). In the serum sample, activated platelets release a variety of metabolites, lipids, and proteases during the coagulation process. Denery et al. reported the detection of more ion features in serum than in plasma, and significantly higher lysophosphatidylinositol signals in plasma [78]. Biochemical studies investigating the difference between these two matrices indicate that serum contains more proteins [79, 80] and has characteristic peaks of peptides and increased levels of hypoxanthine and xanthine [71, 75], thromboxane B2 [76], arginine, and LPCs [73]. Therefore, if serum is used it is of utmost importance to use the same clotting time for all samples in a study and to pay attention to biomarkers that may originate from activated platelets during data evaluation. Of note, the platelet number may vary in the range 20,000–1,000,000 μL^−1^ (reference range: 150,000–450,000 μL^−1^) in a clinical study population. Hence the two most probable, closely related reasons for differences in serum and plasma metabolomes are: first, the activated platelets, which are metabolically very active and release compounds; and second, the need to expose serum tubes to room temperature for proper coagulation [81]. Differences between serum and plasma are summarized in Table 2.

**Table 2:** Compounds that differ between serum and plasma

Different compounds (shown are levels in serum in comparison with plasma)	Refs.
Proteins and peptides Increased: exogenous dipeptide Decreased: fibrinogen	[71, 72]
Lipids Increased: lysophosphatidylcholines (LPCs), diacyl-phosphatidylcholine, thromboxane B2, 12-HHT, 12-HETE, eicosapentaenoic acid, only detected in sera: 11,12-diHETrE, 14,15-diHETrE, 17,18-diHETE Decreased: lysophosphatidylinositols (LPIs), 8-HETE, 15-HETE, 10-HDoHE, 20-HDoHE	[46, 73, 74]
Amino acids Increased: arginine, serine, phenylalanine, glycine, glutamate, cystine phenylalanine, serine, ornithine, proline, methionine, isoleucine, valine, tryptophan	[73–75]
Nucleotides Increased: hypoxanthine, xanthine	[75]
Other metabolites Increased: glycerol-3-phosphate, hydroxybutyrate, ribose, glucose Decreased: pyruvate, citrate, fumarate, glycerate, urate, xylitol	[75–77]

To generate plasma an anticoagulating supplement is needed in the blood-collection tube. The kind of supplement used depends on the diagnostic routine variables to be analyzed. The most common anticoagulants in the clinic are ethylene diamine tetraacetic acid (EDTA), heparin, citrate, and fluoride. Heparin is an anti-thrombin activator, whereas EDTA and citrate chelate calcium ions. EDTA and heparin blood-collection tubes are commonly used in metabolomics studies, although there are still some debates about the best additive for LC–MS-based metabolomics. Actually, the differences in LC–MS analysis between plasma samples generated by different anticoagulants are not evident [71, 82], except for Li^+^-heparin (see above). Of note, for NMR-driven metabolomics analysis EDTA is not recommended because of strong noise signals [83]. For LC–MS applications the cations in the anticoagulants are still a subject of debate, in particular which added cations may cause matrix effects. Barri et al. reported that sodium and potassium formate may cause matrix effects and interferences affecting co-eluting polar metabolites [71]. However, sodium and potassium are the most common cationic additives used in plasma blood-collection tubes. As mentioned above, the presence of Li^+^ may increase the ionization efficiency of many metabolites (Fig. 2), including phospholipids and triacylglycerols [65], but may also increase the signals of plastic polymers and produce serious matrix effects (Fig. 2). Therefore, Li^+^-heparin cannot be recommended for metabolomics analysis. We prefer K^+^–EDTA blood-collection tubes for metabolomics investigations, but as mentioned above, a pretest of the tubes from different companies is absolutely mandatory because the kind of plastic and the composition and purity of additives may vary.

## The effect of hemolysis on the metabolome

Hemolysis is one of the major risks during blood drawing, both in clinical studies and animal experiments [84, 85]. Although it can be avoided by careful drawing and handling of the whole-blood samples, it is the most common preanalytical error in the clinic [86]. As well as strong aspiration there are other causes of hemolysis, including vigorous shaking of the tube, transportation by pneumatic post within the clinic, centrifugation at too high speed, and inadequate environmental temperature. Hemolysis causes the release of intracellular compounds including metabolites and enzymes, which could significantly alter the metabolite profile of the blood sample. Lyses of erythrocytes increase the concentrations of former intracellular metabolites, for example tryptophan, and lipids, for example phospholipids, originating, e.g., from the cellular membrane. Recently we revealed that approximately 18 % of the detected ion mass signals in a non-targeted approach are affected by hemolysis [25].

Hemolytic and non-hemolytic samples can be easily differentiated by their color, because free hemoglobin changes the color of serum or plasma from pale yellow to bright red. However, in slightly hemolytic samples this change in color is not clearly visible, especially when the bilirubin level of the patient is also increased. Free hemoglobin can also be quantitatively measured by a routine two-wavelength clinical chemistry method (reference range: <10 mg dL^−1^), and this is a very reliable method of detecting slight hemolysis compared with qualitative visual evaluation of the sample color. As well as hemolysis, icterus and lipemia are also common preanalytical problems affecting the results of routine clinical tests [87]. In contrast with routine clinical optical assays, icteric and lipemic samples are usually no problem for LC–MS-driven metabolomics analysis.

## The handling of whole blood after drawing

Prolonged exposure of whole blood to room temperature after drawing is another major risk of the preanalytical process. This preanalytical error has the most pronounced effect on the sample quality and consequently on the blood metabolome. Scientists should be aware that a 9 mL tube contains billions of metabolically active blood cells. Therefore, timely separation of serum or plasma from blood cells is mandatory. Of note, because for the generation of serum the whole blood needs to clot at room temperature, the serum metabolite profile may reflect the metabolic action of activated platelets (essential for the coagulation process) as well as the metabolic action of red and white blood cells and the activity of circulating enzymes. Therefore, the clotting time should be strictly controlled and serum samples exposed to different clotting times should not be mixed for metabolomics analysis. According to results from a proteomics study by Timms et al. [88], over-extended clotting time for serum (over 60 min) may lead to cell lyses, whereas a clotting time of less than 30 min leads to incomplete coagulation. An NMR-based metabolomics study confirmed that prolonged clotting time affects the components of the metabolome [89].

**Fig. 3:**
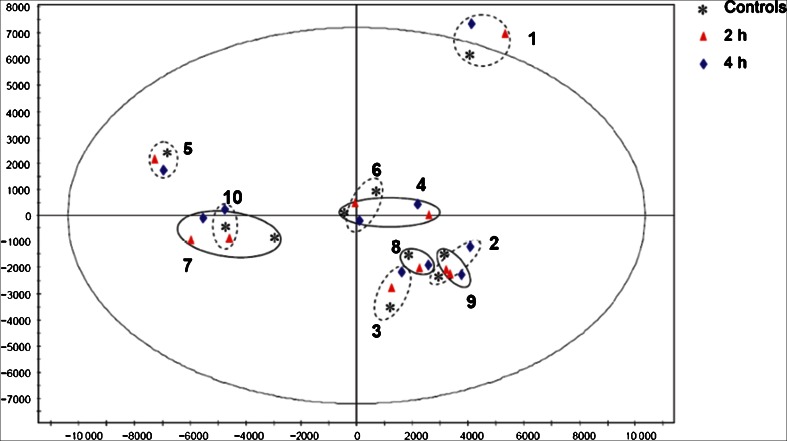
Evaluation of blood samples placed in iced water after blood drawing. The PCA score plot indicated no significant differences between fresh samples and samples placed at once in iced water for 2 or 4 h. (Reprint from Clinical Chemistry (2013) 59 (5):833–845) (License number: 3485201154760)

In contrast with serum-sample tubes, whole blood drawn for the generation of plasma can be put at once into iced water after drawing. Lowered ambient temperature minimizes the metabolic activity of cells and enzymes and keeps the metabolite pattern almost stable. The principle-component-analysis (PCA) score plot given in Fig. 3 reveals no clear difference between whole blood prepared at once (fresh) or after 2 h and 4 h storage in iced water. All samples from the same individual clustered together regardless of the different conditions. These findings from a non-targeted metabolomics approach indicate that only minor changes may occur between freshly prepared plasma sample and whole blood placed on ice for up to 4 h [25]. In an independent study Kamlage et al. replicated these findings using a targeted approach covering 267 metabolites in blood and 262 metabolites in EDTA plasma; the levels of almost all metabolites were stable for up to 6 h in iced water [90]

.

From the practical perspective it should be mentioned that in most clinical studies it is feasible to separate blood cells from plasma within 2 h, and it should be possible in almost all studies to separate the cells from plasma within 4 h. Cooling of whole-blood samples at once in iced water is recommended for short-term preservation before centrifugation and separation of plasma. For the generation of serum a strictly controlled fixed time at room temperature (at least 30 min) followed by immediate cooling is suggested.

## The handling and storage of serum and plasma samples

**Fig. 4:**
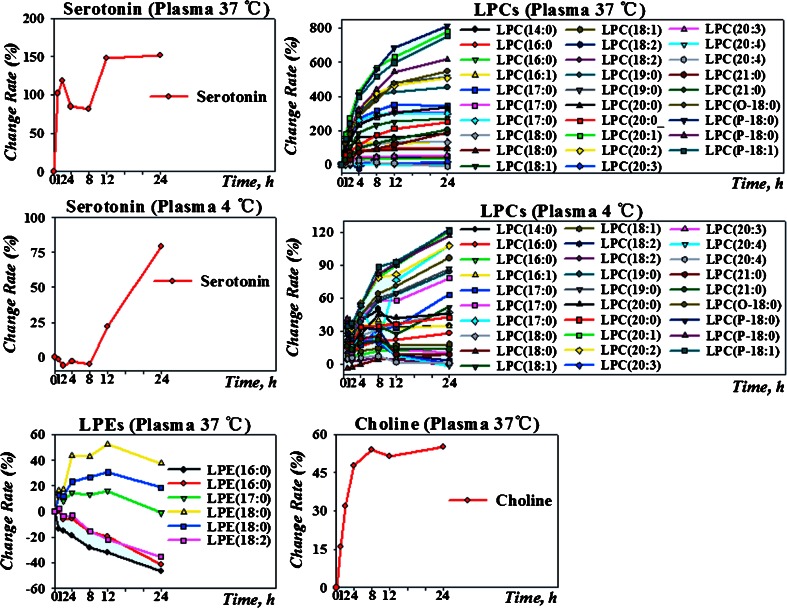
The effect of plasma storage at 37 °C and 4 °C on serotonin, lyso-phospholipids, and choline. LPCs underwent significant changes even at 4 °C. Serotonin and choline were easily affected when placed at 37 °C. Reprinted with permission from Ref. [91] Copyright (2013) American Chemical Society

After the separation of cells the stability of metabolites, and hence of the metabolome, can still be affected by the presence of enzymes and many other proteins in serum and plasma. Hence the handling of plasma or serum is also a relevant preanalytical factor affecting the stability of the metabolome. However, these changes are several orders of magnitude less pronounced than alterations occurring in whole blood in vitro after drawing. Exposure of plasma to room temperature for 16 h resulted in slight, significant changes to 23 % of the analyzed metabolites [91]. Figure 4 shows the effects of 1 h exposure of plasma samples to 37 °C [91]. Plasma stored under these conditions is very unstable; however, the exposure of plasma or serum to 37 °C during sample processing is rather unusual. Prolonged exposure to low temperature, in contrast, causes fewer changes of metabolites. For example, when EDTA plasma is stored for 16 h at 4 °C, only 30 of 262 measured metabolites undergo slight changes [90].

Freezing is the next step in clinical sample handling. Because of the lack of −80 °C freezers in many clinical surroundings, or at least a lack in close proximity, samples are often stored at −20 °C, at least temporarily before a decision is made by the medical doctor as to which properties should be analyzed, whether omics approaches should be used, etc. An NMR-based metabolomics study revealed that storing plasma at −20 °C resulted in significant changes of some metabolites, including glucose and proline [92]. In contrast, according to the results of a clinical chemistry study, 17 common clinical routine analytes in serum, including such metabolites as bilirubin, uric acid, cholesterol, creatinine, and triglycerides, are relatively stable when stored at −20 °C for three months [93]. However, the level of albumin changed significantly. Proteins, in particular albumin, are essential molecules in serum or plasma which absorb and release many small compounds, possibly even in frozen samples. Hence changes in albumin levels may affect the concentration of metabolites. Because of the stability of proteins in serum or plasma, samples can be stored at −70 °C for four years without obvious changes in the concentration [94]. Storage of biofluids at −80 °C or below is regarded as the preferred condition [95]. Pinto et al. found small changes in NMR spectra of plasma metabolites after 20–30 months’ storage at −80 °C [92]; however, low-abundance metabolites, which account for most of the metabolome, are not covered by NMR approaches. Yang et al. reported differences between two sets of plasma stored at −80 °C for two months and for five years [91]. These studies provided a basis for future investigations of the stability of frozen samples.

Because nontargeted metabolomics measures the relative content of thousands of ions, it is difficult to compare the data from different analytical batches. To this end, targeted analysis of metabolites could provide valuable data on sample stability. Hustad et al. studied the stability of B vitamins and metabolites related to one-carbon metabolism in serum samples stored at −25 °C for 29 years [96]. Serum amino acids varied in stability during long-term storage at −25 °C; for example, methionine was transformed to methionine sulfoxide. Furthermore, most B vitamins were found to be unstable during long-term storage. However, other metabolites, including betain, sarcosine, and creatinine, were relatively stable. Metherel et al. studied the stability of eicosapentaenoic acid (EPA) and docosahexaenoic acid (DHA) stored under different conditions [97]. EPA and DHA levels in whole blood are stable for at least 180 days when frozen at −75 °C. At −20 °C a significant reduction of these two polyunsaturated fatty acids was detected [98]. On the basis of current studies, heparin and antioxidant may be a better choice for blood collection to maintain the stability of EPA and DHA during long-term storage [97].

The stability of the entire metabolome under long-term storage conditions is still an open question. There is still a big gap between the recommended ideal storage of body fluids and the reality in daily clinical practice. Although liquid nitrogen might be the condition of choice, it is not feasible and practical in many hospitals, especially in developing countries.

## The effect of freezing and thawing

Serum and plasma are believed to be relatively stable when stored at −80 °C. However, repeated freeze–thaw cycles are unavoidable in clinical studies dealing with a limited number of sample aliquots. In particular, the most valuable sample aliquots are most frequently used to answer novel research questions, which results in rethawing and subsequent refreezing to save this valuable sample material. Stepwise thawing and keeping the samples at 4 °C for as short a time as possible before refreezing is highly recommended. However, usually samples are thawed at room temperature to save time [92, 94, 99–101]. l-Carnitine, lipids, choline phospholipids, alanine, glucose, pyruvate, and acetone changed after four or five freeze–thaw cycles at room temperature [92, 101]. In a clinical-chemistry study, total bilirubin and uric acid were revealed to be unstable even after one or two freeze–thaw cycles at room temperature [92]. However, Comstock et al. reported that cholesterol, micronutrients, and hormones in human plasma did not change after repeated freeze–thaw cycles at room temperature [102]. After four freeze–thaw cycles at 4 °C only 0.5 % (4/706) of metabolite ions changed significantly in our recently published non-targeted metabolomics approach [25]. However, the sensitivity of individual samples to repeated freeze–thaw cycles varies, which led us to conclude that the stability of serum or plasma is also dependent on the donors [25]. Macromolecules including DNA, RNA, and proteins are also affected by frequent refreezing [94, 99]. The DNA yield was reported to decrease by 25 % after one freeze–thaw cycle at room temperature [100]. The metabolite content may be changed by the depositing of protein during freeze–thaw cycles. To avoid potential effects from repeated freeze–thaw cycles it is recommended to divide the samples into small aliquots before storage.

## Sample pretreatment for mass-spectrometry analysis

**Fig. 5:**
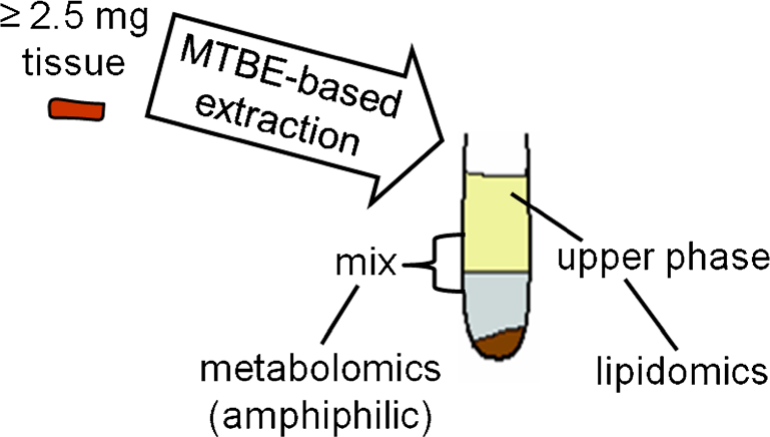
The development of a novel sample-preparation method for metabolomics. Using the solvent system MTBE–methanol–water, polar and non-polar metabolites could be effectively simultaneously extracted from a limited amount of tissue. The method can also be used in the preparation of blood samples. (Reprint from J Chromatogr A 1298:9–16, License number 3526350373512)

Sample pretreatment is the final preanalytical step, and usually takes place in the laboratory of the analytical (bio)chemist. Commonly this step is highly standardized and is less error-prone; however, it is still critical. In particular, inexperienced scientists can be overwhelmed by the huge number of different sample-pretreatment procedures in the literature. The main steps of blood pretreatment for metabolomics include quenching, deproteinization, and extraction. Organic solvents, ultrafiltration, and solid-phase extraction (SPE) are commonly used methods for protein precipitation [103]. In particular, organic solvents including acetonitrile, methanol, chloroform, etc. can be used for quenching and for highly efficient extraction [104] of, e.g., polar metabolites [105] and lipids [106]. Fig. 5 illustrates the strategy using methyl tert-butyl ether (MTBE), methanol, and water to extract metabolites from tissues, which can effectively extract polar and non-polar metabolites simultaneously. This method is also suitable for the preparation of blood samples [106]. Each of these procedures (i.e. organic solvents, ultrafiltration, and SPE) has its strengths but also has limitations, e.g. the loss of one or several subclasses of metabolite as a result of their individual chemical characteristics.

Critical variables for solvent extractions and SPE are the solvent type, pH, and temperature. For SPE the selected solid phase defines which metabolite classes will be reduced or even lost. However, SPE can also be used to support the profiling of low-abundance metabolites by concentrating such compounds and reducing the concentration of high-abundance metabolites.

Keeping the sample pretreatment for mass-spectrometry metabolomics analysis as simple as possible guarantees the highest reproducibility. We suggest deproteinization by the addition of an organic solvent followed by centrifugation under highly standardized conditions, because it is simple and the metabolite coverage is satisfactory. This is a widely accepted, common procedure for metabolomics-sample pretreatment [107]. Another very important, simple, but less respected procedure in this context is to thaw the body fluids in iced water and not at room temperature (see section “The effect of freezing and thawing”). The samples should be kept cooled at all steps to guarantee high sample quality. In general, sample pretreatment for mass-spectrometry analysis is the least critical pre-analytical step because it is performed by analytical experts and can be highly standardized, in contrast with, e.g., blood drawing under highly variable clinical conditions.

## Preanalytical SOP for blood in metabolomics

The continually increasing number of metabolomics applications in clinical research has led to an increasing demand for preanalytical SOPs to standardize the collection, transportation, preparation, and storage of clinical samples [108]. Such SOPs have been commonly used during clinical trials [24] and they are of great importance for targeted and nontargeted clinical metabolomics studies [25, 107, 109]. However, to evaluate the stability of every single ion mass or metabolite detectable by non-targeted metabolomic analysis of blood under all possible clinical conditions is a very challenging task. Therefore, the current preanalytical SOPs are established on the basis of systematic assessments of the acquired data under different preanalytical model conditions, investigating the most critical steps in this complex, error-prone process. As mentioned above, unsupervised modeling, for example PCA, is commonly used to evaluate individual variation of samples. Minor factors will not lead to clustering of samples in the PCA score plot; for example, in Fig. 3 samples from the same donor clustered together, whereas the effect of preanalytical treatment (placing in iced water) was not obvious. According to the nonparametric test and false-discovery-rate (FDR) correction, a few variables will still inevitably undergo statistically significant changes [25]. Therefore, the objective of a practical SOP should be to guarantee the stability of most but not all metabolites of the metabolome (e.g. ≥98 %), which should be achievable. Suggestions for an SOP can be found in Ref. [25].

## Conclusions

In summary, the preanalytical procedures of blood sampling and processing may have apparent and strong effects on the results of metabolomics studies. Taking into account the variance in the population, preanalytical aspects include several steps, from study design to sample collection, transportation, and storage. The preanalytical challenges for metabolomics of blood are mainly caused by two factors: the stability of metabolites and the metabolic action of cells in the sample collection tube. Therefore, SOPs describing the preanalytical process must take into account all possible aspects, including, e.g., the choice of tubes and tips. Because metabolomics always focuses on the changes after biological perturbation, it is of great importance that the preanalytical processes for all enrolled samples are performed as accurately as possible. Studies focused on preanalytical problems are the basis for recommendations and SOPs to achieve comparable and reproducible results in metabolomics studies. The principles can be briefly summarized as follows:

1. For pilot metabolomics studies analyzing a limited number of samples, carefully considered study design is mandatory.
2. Before the start of a study the plastic blood-collection tubes and plastic pipette tips (for sample pipetting) intended for use must pass a chemical-noise test by MS analysis.
3. Hemolytic samples, which can be identified by the levels of free hemoglobin, should be excluded from a study.
4. Collected blood samples (for plasma generation) should be placed at once in iced water after drawing, and blood intended for serum preparation should be placed in iced water immediately after 30 min clotting at room temperature.
5. Prompt (within 30 min) separation of blood cells from plasma by centrifugation at 4 °C is ideal, but storage of whole blood for a maximum of 4 h in iced water before centrifugation is acceptable and should be feasible in almost all clinical studies.
6. Samples should be stored at −80 °C or below. Stepwise freezing is recommended.
7. Repeated freeze–thaw cycles should be avoided.
8. An individual SOP for the sample pretreatment for mass-spectrometry analysis is recommended, because this pre-analytical step is not performed at once, e.g. in the clinic, but in the laboratory of the analytical (bio)chemists.

## References

[1] Nicholson JK, Lindon JC, Holmes E (1999). 'Metabonomics': understanding the metabolic responses of living systems to pathophysiological stimuli via multivariate statistical analysis of biological NMR spectroscopic data. Xenobiotica.

[2] Sreekumar A, Poisson LM, Rajendiran TM, Khan AP, Cao Q, Yu JD, Laxman B, Mehra R, Lonigro RJ, Li Y, Nyati MK, Ahsan A, Kalyana-Sundaram S, Han B, Cao XH, Byun J, Omenn GS, Ghosh D, Pennathur S, Alexander DC, Berger A, Shuster JR, Wei JT, Varambally S, Beecher C, Chinnaiyan AM (2009). Metabolomic profiles delineate potential role for sarcosine in prostate cancer progression. Nature.

[3] Lokhov PG, Dashtiev MI, Moshkovskii SA, Archakov AI (2010). Metabolite profiling of blood plasma of patients with prostate cancer. Metabolomics.

[4] Huang Q, Tan YX, Yin PY, Ye GZ, Gao P, Lu X, Wang HY, Xu GW (2013). Metabolic Characterization of Hepatocellular Carcinoma Using Nontargeted Tissue Metabolomics. Cancer Res.

[5] Duarte IF, Rocha CM, Gil AM (2013). Metabolic profiling of biofluids: potential in lung cancer screening and diagnosis. Expert Rev Mol Diagn.

[6] Vuckovic D (2012). Current trends and challenges in sample preparation for global metabolomics using liquid chromatography-mass spectrometry. Anal Bioanal Chem.

[7] Kinross JM, Holmes E, Darzi AW, Nicholson JK (2011). Metabolic phenotyping for monitoring surgical patients. Lancet.

[8] Wei J, Xie G, Zhou Z, Shi P, Qiu Y, Zheng X, Chen T, Su M, Zhao A, Jia W (2011). Salivary metabolite signatures of oral cancer and leukoplakia. Int J Cancer.

[9] Cuevas-Cordoba B, Santiago-Garcia J (2014). Saliva: a fluid of study for OMICS. OMICS.

[10] Alvarez-Sanchez B, Priego-Capote F, Luque de Castro MD (2012). Study of sample preparation for metabolomic profiling of human saliva by liquid chromatography-time of flight/mass spectrometry. J Chromatogr A.

[11] Ibanez C, Simo C, Barupal DK, Fiehn O, Kivipelto M, Cedazo-Minguez A, Cifuentes A (2013). A new metabolomic workflow for early detection of Alzheimer's disease. J Chromatogr A.

[12] Trushina E, Dutta T, Persson XM, Mielke MM, Petersen RC (2013). Identification of altered metabolic pathways in plasma and CSF in mild cognitive impairment and Alzheimer's disease using metabolomics. PLoS One.

[13] Kaddurah-Daouk R, Yuan P, Boyle SH, Matson W, Wang Z, Zeng ZB, Zhu H, Dougherty GG, Yao JK, Chen G, Guitart X, Carlson PJ, Neumeister A, Zarate C, Krishnan RR, Manji HK, Drevets W (2012). Cerebrospinal fluid metabolome in mood disorders-remission state has a unique metabolic profile. Sci Rep.

[14] Le Gall G, Noor SO, Ridgway K, Scovell L, Jamieson C, Johnson IT, Colquhoun IJ, Kemsley EK, Narbad A (2011). Metabolomics of fecal extracts detects altered metabolic activity of gut microbiota in ulcerative colitis and irritable bowel syndrome. J Proteome Res.

[15] Goedert JJ, Sampson JN, Moore SC, Xiao Q, Xiong X, Hayes RB, Ahn J, Shi J, Sinha R (2014). Fecal metabolomics: assay performance and association with colorectal cancer. Carcinogenesis.

[16] Ng Hublin JS, Ryan U, Trengove R, Maker G (2013). Metabolomic profiling of faecal extracts from Cryptosporidium parvum infection in experimental mouse models. PLoS One.

[17] O'Gorman A, Wallace M, Cottell E, Gibney MJ, McAuliffe FM, Wingfield M, Brennan L (2013). Metabolic profiling of human follicular fluid identifies potential biomarkers of oocyte developmental competence. Reproduction.

[18] Revelli A, Delle Piane L, Casano S, Molinari E, Massobrio M, Rinaudo P (2009). Follicular fluid content and oocyte quality: from single biochemical markers to metabolomics. Reprod Biol Endocrinol.

[19] Bouatra S, Aziat F, Mandal R, Guo AC, Wilson MR, Knox C, Bjorndahl TC, Krishnamurthy R, Saleem F, Liu P, Dame ZT, Poelzer J, Huynh J, Yallou FS, Psychogios N, Dong E, Bogumil R, Roehring C, Wishart DS (2013). The human urine metabolome. PLoS One.

[20] Kaddurah-Daouk R, Kristal BS, Weinshilboum RM (2008). Metabolomics: a global biochemical approach to drug response and disease. Annu Rev Pharmacol Toxicol.

[21] Gibney MJ, Walsh M, Brennan L, Roche HM, German B, van Ommen B (2005). Metabolomics in human nutrition: opportunities and challenges. Am J Clin Nutr.

[22] Wishart DS (2008). Applications of metabolomics in drug discovery and development. Drugs R D.

[23] Holland NT, Pfleger L, Berger E, Ho A, Bastaki M (2005). Molecular epidemiology biomarkers - Sample collection and processing considerations. Toxicol Appl Pharm.

[24] Leyland-Jones BR, Ambrosone CB, Bartlett J, Ellis MJ, Enos RA, Raji A, Pins MR, Zujewski JA, Hewitt SM, Forbes JF, Abramovitz M, Braga S, Cardoso F, Harbeck N, Denkert C, Jewell SD, Breast International G, Cooperative Groups of the Breast Cancer Intergroup of North A, American College of Surgeons Oncology G, Cancer, Leukemia Group B, Eastern Cooperative Oncology G, North Central Cancer Treatment G, National Cancer Institute of Canada Clinical Trials G, Southwest Oncology G, National Surgical Adjuvant B, Bowel P, Radiation Oncology G, Gynecologic Oncology G, Children's Oncology G (2008). Recommendations for collection and handling of specimens from group breast cancer clinical trials. J Clin Oncol.

[25] Yin P, Peter A, Franken H, Zhao X, Neukamm SS, Rosenbaum L, Lucio M, Zell A, Haring HU, Xu G, Lehmann R (2013). Preanalytical aspects and sample quality assessment in metabolomics studies of human blood. Clin Chem.

[26] Guder WG (2014). History of the preanalytical phase: a personal view. Biochemia Med.

[27] Ueland PM, Refsum H, Stabler SP, Malinow MR, Andersson A, Allen RH (1993). Total homocysteine in plasma or serum: methods and clinical applications. Clin Chem.

[28] Cooper GR, Myers GL, Smith SJ, Sampson EJ (1988). Standardization of lipid, lipoprotein, and apolipoprotein measurements. Clin Chem.

[29] Delanghe J, Speeckaert M (2014). Preanalytical requirements of urinalysis. Biochemia Med.

[30] Ament Z, Masoodi M, Griffin JL (2012). Applications of metabolomics for understanding the action of peroxisome proliferator-activated receptors (PPARs) in diabetes, obesity and cancer. Genome Med.

[31] van der Greef J, van Wietmarschen H, van Ommen B, Verheij E (2013). Looking back into the future: 30 years of metabolomics at TNO. Mass Spectrom Rev.

[32] Griffin JL, Atherton H, Shockcor J, Atzori L (2011). Metabolomics as a tool for cardiac research. Nat Rev Cardiol.

[33] Abu Aboud O, Weiss RH (2013). New Opportunities from the Cancer Metabolome. Clin Chem.

[34] Spratlin JL, Serkova NJ, Eckhardt SG (2009). Clinical Applications of Metabolomics in Oncology: A Review. Clin Cancer Res.

[35] Wang TJ, Larson MG, Vasan RS, Cheng S, Rhee EP, McCabe E, Lewis GD, Fox CS, Jacques PF, Fernandez C, O'Donnell CJ, Carr SA, Mootha VK, Florez JC, Souza A, Melander O, Clish CB, Gerszten RE (2011). Metabolite profiles and the risk of developing diabetes. Nat Med.

[36] Yin P, Xu G (2013). Metabolomics for tumor marker discovery and identification based on chromatography-mass spectrometry. Expert Rev Mol Diagn.

[37] Chen J, Zhang XY, Cao R, Lu X, Zhao SM, Fekete A, Huang Q, Schmitt-Kopplin P, Wang YS, Xu ZL, Wan XP, Wu XH, Zhao NQ, Xu CJ, Xu GW (2011). Serum 27-nor-5 beta-Cholestane-3,7,12,24,25 Pentol Glucuronide Discovered by Metabolomics as Potential Diagnostic Biomarker for Epithelium Ovarian Cancer. J Proteome Res.

[38] Gika H, Theodoridis G (2011). Sample preparation prior to the LC-MS-based metabolomics/metabonomics of blood-derived samples. Bioanalysis.

[39] Bijlsma S, Bobeldijk I, Verheij ER, Ramaker R, Kochhar S, Macdonald IA, van Ommen B, Smilde AK (2006). Large-scale human metabolomics studies: a strategy for data (pre-) processing and validation. Anal Chem.

[40] Eichner J, Rosenbaum L, Wrzodek C, Haring H-U, Zell A, Lehmann R (2014). Integrated enrichment analysis and pathway-centered visualization of metabolomics, proteomics, transcriptomics, and genomics data by using the InCroMAP software. J Chromatogr B Analyt Technol Biomed Life Sci.

[41] Minami Y, Kasukawa T, Kakazu Y, Iigo M, Sugimoto M, Ikeda S, Yasui A, van der Horst GT, Soga T, Ueda HR (2009). Measurement of internal body time by blood metabolomics. Proc Natl Acad Sci U S A.

[42] Weigert C, Lehmann R, Hartwig S, Lehr S (2014). The secretome of the working human skeletal muscle-a promising opportunity to combat the metabolic disaster?. Proteomics Clin Appl.

[43] Griffin JL, Bollard ME (2004). Metabonomics: its potential as a tool in toxicology for safety assessment and data integration. Curr Drug Metab.

[44] Narayanan S (2000). The preanalytic phase. An important component of laboratory medicine. Am J Clin Pathol.

[45] Ishikawa M, Maekawa K, Saito K, Senoo Y, Urata M, Murayama M, Tajima Y, Kumagai Y, Saito Y (2014). Plasma and serum lipidomics of healthy white adults shows characteristic profiles by subjects' gender and age. PLoS One.

[46] Ishikawa M, Tajima Y, Murayama M, Senoo Y, Maekawa K, Saito Y (2013). Plasma and serum from nonfasting men and women differ in their lipidomic profiles. Biol Pharm Bull.

[47] Lawton KA, Berger A, Mitchell M, Milgram KE, Evans AM, Guo L, Hanson RW, Kalhan SC, Ryals JA, Milburn MV (2008). Analysis of the adult human plasma metabolome. Pharmacogenomics.

[48] Chan YC, Suzuki M, Yamamoto S (1999). A comparison of anthropometry, biochemical variables and plasma amino acids among centenarians, elderly and young subjects. J Am Coll Nutr.

[49] Morris C, O'Grada C, Ryan M, Roche HM, Gibney MJ, Gibney ER, Brennan L (2012). The relationship between BMI and metabolomic profiles: a focus on amino acids. Proc Nutr Soc.

[50] Kochhar S, Jacobs DM, Ramadan Z, Berruex F, Fuerholz A, Fay LB (2006). Probing gender-specific metabolism differences in humans by nuclear magnetic resonance-based metabonomics. Anal Biochem.

[51] Newgard CB, An J, Bain JR, Muehlbauer MJ, Stevens RD, Lien LF, Haqq AM, Shah SH, Arlotto M, Slentz CA, Rochon J, Gallup D, Ilkayeva O, Wenner BR, Yancy WS, Eisenson H, Musante G, Surwit RS, Millington DS, Butler MD, Svetkey LP (2009). A branched-chain amino acid-related metabolic signature that differentiates obese and lean humans and contributes to insulin resistance. Cell Metab.

[52] Lucio M, Fekete A, Weigert C, Wagele B, Zhao X, Chen J, Fritsche A, Haring H-U, Schleicher ED, Xu G, Schmitt-Kopplin P, Lehmann R (2010). Insulin sensitivity is reflected by characteristic metabolic fingerprints–a Fourier transform mass spectrometric non-targeted metabolomics approach. PLoS One.

[53] Brauer R, Leichtle AB, Fiedler GM, Thiery J, Ceglarek U (2011). Preanalytical standardization of amino acid and acylcarnitine metabolite profiling in human blood using tandem mass spectrometry. Metabolomics.

[54] Simundic AM, Cornes M, Grankvist K, Lippi G, Nybo M (2014). Standardization of collection requirements for fasting samples: For the Working Group on Preanalytical Phase (WG-PA) of the European Federation of Clinical Chemistry and Laboratory Medicine (EFLM). Clin Chim Acta.

[55] Gooley JJ, Chua EC (2014). Diurnal Regulation of Lipid Metabolism and Applications of Circadian Lipidomics. J Genet Genomics.

[56] Ang JE, Revell V, Mann A, Mantele S, Otway DT, Johnston JD, Thumser AE, Skene DJ, Raynaud F (2012). Identification of human plasma metabolites exhibiting time-of-day variation using an untargeted liquid chromatography-mass spectrometry metabolomic approach. Chronobiol Int.

[57] Kasukawa T, Sugimoto M, Hida A, Minami Y, Mori M, Honma S, Honma K-i, Mishima K, Soga T, Ueda HR (2012). Human blood metabolite timetable indicates internal body time. Proc Natl Acad Sci U S A.

[58] Lehmann R, Zhao X, Weigert C, Simon P, Fehrenbach E, Fritsche J, Machann J, Schick F, Wang J, Hoene M, Schleicher ED, Haring HU, Xu G, Niess AM (2010). Medium chain acylcarnitines dominate the metabolite pattern in humans under moderate intensity exercise and support lipid oxidation. PLoS One.

[59] Slupsky CM, Rankin KN, Wagner J, Fu H, Chang D, Weljie AM, Saude EJ, Lix B, Adamko DJ, Shah S, Greiner R, Sykes BD, Marrie TJ (2007). Investigations of the effects of gender, diurnal variation, and age in human urinary metabolomic profiles. Anal Chem.

[60] Gillio-Meina C, Cepinskas G, Cecchini EL, Fraser DD (2013). Translational research in pediatrics II: blood collection, processing, shipping, and storage. Pediatrics.

[61] Winnike JH, Busby MG, Watkins PB, O'Connell TM (2009). Effects of a prolonged standardized diet on normalizing the human metabolome. Am J Clin Nutr.

[62] Pechlivanis A, Kostidis S, Saraslanidis P, Petridou A, Tsalis G, Veselkov K, Mikros E, Mougios V, Theodoridis GA (2013). 1H NMR study on the short- and long-term impact of two training programs of sprint running on the metabolic fingerprint of human serum. J Proteome Res.

[63] Hsu PC, Zhou B, Zhao Y, Ressom HW, Cheema AK, Pickworth W, Shields PG (2013). Feasibility of identifying the tobacco-related global metabolome in blood by UPLC-QTOF-MS. J Proteome Res.

[64] Dallmann R, Viola AU, Tarokh L, Cajochen C, Brown SA (2012). The human circadian metabolome. Proc Natl Acad Sci U S A.

[65] Mei H, Hsieh Y, Nardo C, Xu X, Wang S, Ng K, Korfmacher WA (2003). Investigation of matrix effects in bioanalytical high-performance liquid chromatography/tandem mass spectrometric assays: application to drug discovery. Rapid Commun Mass Spectrom.

[66] Koulman A, Prentice P, Wong MC, Matthews L, Bond NJ, Eiden M, Griffin JL, Dunger DB (2014). The development and validation of a fast and robust dried blood spot based lipid profiling method to study infant metabolism. Metabolomics.

[67] Kong ST, Lin HS, Ching J, Ho PC (2011). Evaluation of Dried Blood Spots as Sample Matrix for Gas Chromatography/Mass Spectrometry Based Metabolomic Profiling. Anal Chem.

[68] Gucciardi A, Pirillo P, Di Gangi IM, Naturale M, Giordano G (2012). A rapid UPLC-MS/MS method for simultaneous separation of 48 acylcarnitines in dried blood spots and plasma useful as a second-tier test for expanded newborn screening. Anal Bioanal Chem.

[69] Michopoulos F, Theodoridis G, Smith CJ, Wilson ID (2010). Metabolite Profiles from Dried Biofluid Spots for Metabonomic Studies using UPLC Combined with oaToF-MS. J Proteome Res.

[70] Michopoulos F, Theodoridis G, Smith CJ, Wilson ID (2011). Metabolite profiles from dried blood spots for metabonomic studies using UPLC combined with orthogonal acceleration ToF-MS: effects of different papers and sample storage stability. Bioanalysis.

[71] Barri T, Dragsted LO (2013). UPLC-ESI-QTOF/MS and multivariate data analysis for blood plasma and serum metabolomics: effect of experimental artefacts and anticoagulant. Anal Chim Acta.

[72] Ladenson JH, Tsai LM, Michael JM, Kessler G, Joist JH (1974). Serum versus heparinized plasma for eighteen common chemistry tests: is serum the appropriate specimen?. Am J Clin Pathol.

[73] Yu Z, Kastenmuller G, He Y, Belcredi P, Moller G, Prehn C, Mendes J, Wahl S, Roemisch-Margl W, Ceglarek U, Polonikov A, Dahmen N, Prokisch H, Xie L, Li Y, Wichmann HE, Peters A, Kronenberg F, Suhre K, Adamski J, Illig T, Wang-Sattler R (2011). Differences between human plasma and serum metabolite profiles. PLoS One.

[74] Lin Z, Zhang Z, Lu H, Jin Y, Yi L, Liang Y (2014). Joint MS-based platforms for comprehensive comparison of rat plasma and serum metabolic profiling. Biomed Chromatogr.

[75] Liu L, Aa J, Wang G, Yan B, Zhang Y, Wang X, Zhao C, Cao B, Shi J, Li M, Zheng T, Zheng Y, Hao G, Zhou F, Sun J, Wu Z (2010). Differences in metabolite profile between blood plasma and serum. Anal Biochem.

[76] Wedge DC, Allwood JW, Dunn W, Vaughan AA, Simpson K, Brown M, Priest L, Blackhall FH, Whetton AD, Dive C, Goodacre R (2011). Is serum or plasma more appropriate for intersubject comparisons in metabolomic studies? An assessment in patients with small-cell lung cancer. Anal Chem.

[77] Dettmer K, Almstetter MF, Appel IJ, Nurnberger N, Schlamberger G, Gronwald W, Meyer HH, Oefner PJ (2010). Comparison of serum versus plasma collection in gas chromatography–mass spectrometry-based metabolomics. Electrophoresis.

[78] Denery JR, Nunes AAK, Dickerson TJ (2011). Characterization of differences between blood sample matrices in untargeted metabolomics. Anal Chem.

[79] Barelli S, Crettaz D, Thadikkaran L, Rubin O, Tissot J-D (2007). Plasma/serum proteomics: pre-analytical issues. Expert Rev Proteomics.

[80] Issaq HJ, Xiao Z, Veenstra TD (2007). Serum and plasma proteomics. Chem Rev.

[81] Wung WE, Howell SB (1980). Simultaneous liquid chromatography of 5-fluorouracil, uridine, hypoxanthine, xanthine, uric acid, allopurinol, and oxipurinol in plasma. Clin Chem.

[82] Denery JR, Nunes AA, Dickerson TJ (2011). Characterization of differences between blood sample matrices in untargeted metabolomics. Anal Chem.

[83] Nicholson JK, Buckingham MJ, Sadler PJ (1983). High resolution 1H n.m.r. studies of vertebrate blood and plasma. Biochem J.

[84] Theil PK, Pedersen LJ, Jensen MB, Yde CC, Bach Knudsen KE (2012). Blood sampling and hemolysis affect concentration of plasma metabolites. J Anim Sci.

[85] Agarwal S, Vargas G, Nordstrom C, Tam E, Buffone GJ, Devaraj S (2014). Effect of interference from hemolysis, icterus and lipemia on routine pediatric clinical chemistry assays. Clin Chim Acta.

[86] Gimenez-Marin A, Rivas-Ruiz F, Perez-Hidalgo Mdel M, Molina-Mendoza P (2014). Pre-analytical errors management in the clinical laboratory: a five-year study. Biochem Med (Zagreb).

[87] Ji JZ, Meng QH (2011). Evaluation of the interference of hemoglobin, bilirubin, and lipids on Roche Cobas 6000 assays. Clin Chim Acta.

[88] Timms JF, Arslan-Low E, Gentry-Maharaj A, Luo Z, T'Jampens D, Podust VN, Ford J, Fung ET, Gammerman A, Jacobs I, Menon U (2007). Preanalytic influence of sample handling on SELDI-TOF serum protein profiles. Clin Chem.

[89] Teahan O, Gamble S, Holmes E, Waxman J, Nicholson JK, Bevan C, Keun HC (2006). Impact of analytical bias in metabonomic studies of human blood serum and plasma. Anal Chem.

[90] Kamlage B, Maldonado SG, Bethan B, Peter E, Schmitz O, Liebenberg V, Schatz P (2014). Quality markers addressing preanalytical variations of blood and plasma processing identified by broad and targeted metabolite profiling. Clin Chem.

[91] Yang W, Chen Y, Xi C, Zhang R, Song Y, Zhan Q, Bi X, Abliz Z (2013). Liquid chromatography-tandem mass spectrometry-based plasma metabonomics delineate the effect of metabolites' stability on reliability of potential biomarkers. Anal Chem.

[92] Pinto J, Domingues MR, Galhano E, Pita C, Almeida MD, Carreira IM, Gil AM (2014). Human plasma stability during handling and storage: impact on NMR metabolomics. Analyst.

[93] Cuhadar S, Koseoglu M, Atay A, Dirican A (2013). The effect of storage time and freeze-thaw cycles on the stability of serum samples. Biochem Med (Zagreb).

[94] Mitchell BL, Yasui Y, Li CI, Fitzpatrick AL, Lampe PD (2005). Impact of freeze-thaw cycles and storage time on plasma samples used in mass spectrometry based biomarker discovery projects. Cancer Informat.

[95] Vaught JB (2006). Blood collection, shipment, processing, and storage. Cancer Epidemiol Biomarkers Prev.

[96] Hustad S, Eussen S, Midttun O, Ulvik A, van de Kant PM, Morkrid L, Gislefoss R, Ueland PM (2012). Kinetic modeling of storage effects on biomarkers related to B vitamin status and one-carbon metabolism. Clin Chem.

[97] Metherel AH, Henao JJA, Stark KD (2013). EPA and DHA Levels in Whole Blood Decrease More Rapidly when Stored at -20 degrees C as Compared with Room Temperature, 4 and -75 degrees C. Lipids.

[98] Pottala JV, Espeland MA, Polreis J, Robinson J, Harris WS (2012). Correcting the effects of -20 degrees C storage and aliquot size on erythrocyte fatty acid content in the Women's Health Initiative. Lipids.

[99] Rai AJ, Stemmer PM, Zhang Z, Adam B-L, Morgan WT, Caffrey RE, Podust VN, Patel M, Lim L-Y, Shipulina NV, Chan DW, Semmes OJ, Leung H-CE (2005). Analysis of Human Proteome Organization Plasma Proteome Project (HUPO PPP) reference specimens using surface enhanced laser desorption/ionization-time of flight (SELDI-TOF) mass spectrometry: multi-institution correlation of spectra and identification of biomarkers. Proteomics.

[100] Ross KS, Haites NE, Kelly KF (1990). Repeated freezing and thawing of peripheral blood and DNA in suspension: effects on DNA yield and integrity. J Med Genet.

[101] Fliniaux O, Gaillard G, Lion A, Cailleu D, Mesnard F, Betsou F (2011). Influence of common preanalytical variations on the metabolic profile of serum samples in biobanks. J Biomol NMR.

[102] Comstock GW, Burke AE, Norkus EP, Gordon GB, Hoffman SC, Helzlsouer KJ (2001). Effects of repeated freeze-thaw cycles on concentrations of cholesterol, micronutrients, and hormones in human plasma and serum. Clin Chem.

[103] Michopoulos F, Lai L, Gika H, Theodoridis G, Wilson I (2009). UPLC-MS-Based Analysis of Human Plasma for Metabonomics Using Solvent Precipitation or Solid Phase Extraction. J Proteome Res.

[104] Want EJ (2006). Solvent-dependent metabolite distribution, clustering, and protein extraction for serum profiling with mass spectrometry. Anal Chem.

[105] Gika HG, Theodoridis GA, Wilson ID (2008). Hydrophilic interaction and reversed-phase ultra-performance liquid chromatography TOF-MS for metabonomic analysis of Zucker rat urine. J Sep Sci.

[106] Chen S, Hoene M, Li J, Li Y, Zhao X, Haring HU, Schleicher ED, Weigert C, Xu G, Lehmann R (2013). Simultaneous extraction of metabolome and lipidome with methyl tert-butyl ether from a single small tissue sample for ultra-high performance liquid chromatography/mass spectrometry. J Chromatogr A.

[107] Dunn WB, Broadhurst D, Begley P, Zelena E, Francis-McIntyre S, Anderson N, Brown M, Knowles JD, Halsall A, Haselden JN, Nicholls AW, Wilson ID, Kell DB, Goodacre R (2011). Procedures for large-scale metabolic profiling of serum and plasma using gas chromatography and liquid chromatography coupled to mass spectrometry. Nat Protoc.

[108] Leichtle AB, Dufour JF, Fiedler GM (2013). Potentials and pitfalls of clinical peptidomics and metabolomics. Swiss Med Wkly.

[109] Helmschrodt C, Becker S, Thiery J, Ceglarek U (2014). Preanalytical standardization for reactive oxygen species derived oxysterol analysis in human plasma by liquid chromatography-tandem mass spectrometry. Biochem Biophys Res Commun.

